# Water‐Coupled Interfacial Modulation Enhances Photovoltaic and Pyroelectric Responses in Transparent Self‐Powered ZnO/NiO Photodetectors

**DOI:** 10.1002/advs.78373

**Published:** 2026-10-22

**Authors:** Seunghee Cho, Vivek Mohan More, Sanh Vo Thi, Md Arifur Rahman Barno, Seojun Jeon, Yebean Cho, Joondong Kim

**Affiliations:** ^1^ Photoelectric and Energy Device Application Lab (PEDAL) Multidisciplinary Core Institute for Future Energies (MCIFE) Incheon National University Incheon Republic of Korea; ^2^ Department of Electrical Engineering Incheon National University Incheon Republic of Korea

**Keywords:** electric double layer, pyroelectric response, self‐powered photodetection, transparent photodetector, water contact, ZnO/NiO heterojunction

## Abstract

Transparent self‐powered photodetectors are attractive for smart windows, environmental monitoring, and underwater optoelectronics, yet using the surrounding medium remains largely unexplored. Here, we demonstrate a water‐assisted interfacial modulation strategy in a transparent FTO/ZnO/NiO/AgNW/ZnO photodetector, where direct water contact enhances performance. Water introduces two interfacial effects. First, refractive‐index matching at the ZnO/water interface suppresses Fresnel reflection, raising average visible transmittance from 61.0% to 77.02%. Second, an electric double layer (EDL) electrostatically modulates the ZnO surface, promoting charge separation and carrier transport. Under 365 nm illumination, the open‐circuit voltage (*V_oc_*) increases from 0.223 to 0.265 V, the short‐circuit current density from 0.751 to 0.797 mA cm^−2^, and the power conversion efficiency from 1.63% to 2.53%. Impedance and Mott–Schottky analyses confirm reduced series resistance, enhanced interfacial capacitance, and a positive flat‐band shift. The water‐induced interfacial field strengthens transient polarization, yielding a responsivity of 93.1 mA W^−1^, a specific detectivity of 1.52 × 10^11^ Jones, an effective pyroelectric coefficient rising from 6.8 to 8.5 mC m^−2^ K^−1^, and fast rise/fall times of 0.953/2.82 ms. These findings establish liquid‐interface engineering as an effective and reversible strategy for simultaneously enhancing transparency and photodetection, opening new opportunities for water‐compatible transparent electronics, intelligent glazing, and submerged optical sensing.

## Graphical abstract

Direct water contact enhances the performance of a transparent self‐powered FTO/ZnO/NiO/AgNW/ZnO UV photodetector. Reduced surface reflection improves optical transmission, while an electric double layer at the ZnO/water interface modulates interfacial electrostatics and promotes charge separation. Combined optical, electrostatic, and thermal effects enhance photovoltaic output, pyroelectric current, responsivity, and detectivity compared with air operation, supporting water‐compatible photoelectric applications.

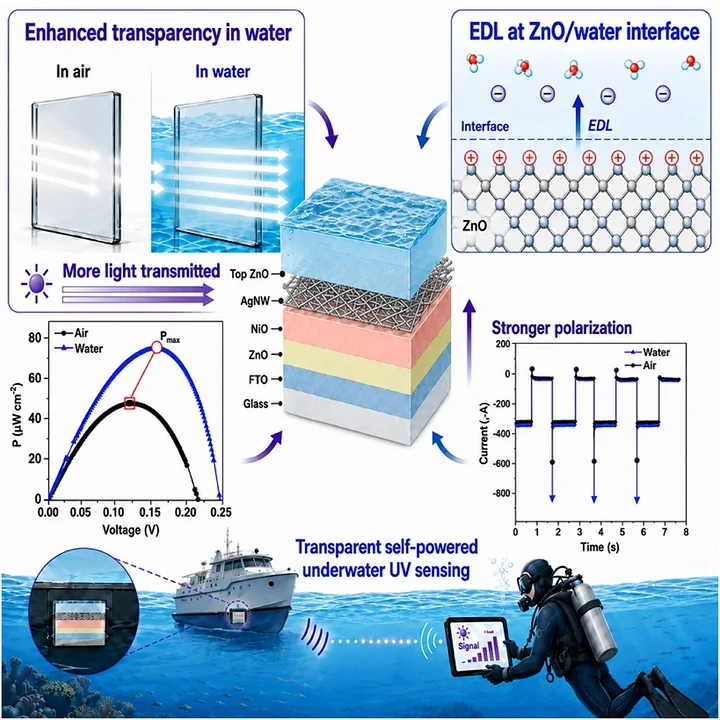

**Abbreviations**
FTO: fluorine‐doped tin oxide
AgNW: silver nanowire
TPV: transparent photovoltaic
TCE: transparent conductive electrode
AVT: average visible transmittance
EDL: electric double layer
EIS: electrochemical impedance spectroscopy
CPE: constant‐phase element
PCE: power conversion efficiency
FF: fill factor
DI: deionized water
LED: light‐emitting diode
TEM: transmission electron microscopy
FETEM: field‐emission transmission electron microscopy
UV: ultraviolet
UV–vis–NIR: ultraviolet–visible–near‐infrared
RF: radio frequency
DC: direct current
V_FB_
: flat‐band potential
NR: not reported
AM 1.5G: Air Mass 1.5 Global

## 1 Introduction

Photodetectors are important optoelectronic devices that convert incident light into electrical signals and are widely used in optical communication, imaging, biomedical monitoring, environmental sensing, and integrated electronic systems [1, 2, 3, 4, 5]. With the increasing demand for low‐power, wireless, and autonomous technologies, self‐powered photodetectors that operate without an external bias have attracted considerable attention [6, 7, 8, 9, 10]. Among them, transparent self‐powered photodetectors are especially promising for smart windows, transparent displays, wearable electronics, and building‐integrated sensing systems, where light detection must be achieved without blocking visible transmission [11, 12, 13, 14, 15]. In such systems, transparency is not only an optical requirement but also a critical factor for practical integration with multifunctional energy and sensing platforms [16, 17].

A common strategy for realizing self‐powered photodetection is to employ a photovoltaic junction as the internal driving source [17, 18]. In this configuration, the built‐in electric field at the junction separates photogenerated electrons and holes, enabling photocurrent generation under zero‐bias conditions [9, 10]. Transparent photovoltaic‐type photodetectors are therefore attractive because they can combine optical transparency, light‐energy conversion, and photodetection in a single device platform [11, 16, 17]. However, their photoresponse can still be limited by insufficient carrier separation, interfacial recombination, and weak transient current generation under low‐intensity or pulsed illumination. Therefore, improving the steady and transient photoresponse while preserving transparency and structural simplicity remains an important challenge [20, 21].

Wide‐bandgap oxide semiconductors provide a useful materials platform for this purpose because they exhibit low visible‐light absorption, good environmental stability, and suitable electronic properties for transparent junction formation [22, 23]. In this work, we demonstrate a transparent self‐powered photodetector based on an FTO/ZnO/NiO/AgNW/ZnO heterojunction. The device is designed to integrate visible‐light transparency, photovoltaic carrier separation, and pyroelectric transient response. In this structure, FTO acts as the transparent bottom electrode, ZnO serves as the main photoactive and electron‐transporting layer, NiO functions as the hole‐transporting layer, and the AgNW/ZnO electrode enables transparent current collection. Because ZnO has a bandgap of approximately 3.3 eV and NiO typically exhibits a wider bandgap of about 3.6–4.0 eV, both layers allow visible‐light transmission while forming an oxide p–n heterojunction [22, 23, 24, 25, 26]. The built‐in electric field at the ZnO/NiO interface supports photogenerated carrier separation, allowing the device to operate without an external bias [27, 28].

ZnO is particularly important because it contributes not only to photovoltaic charge transport but also to transient pyroelectric response. Owing to its non‐centrosymmetric wurtzite crystal structure, ZnO can generate polarization variation under transient temperature changes [29, 30, 31, 32]. Under pulsed illumination, light absorption can induce periodic thermal fluctuation, which changes the pyroelectric polarization and produces a transient current component [33, 34]. Therefore, the total self‐powered photoresponse can include both a steady photovoltaic current generated by photocarriers and a transient pyroelectric current associated with illumination‐induced temperature modulation. The theoretical mechanism of this pyroelectric response is illustrated in Supplementary Figure S1. This photovoltaic–pyroelectric coupling is beneficial because the ZnO/NiO junction provides zero‐bias carrier separation, while the pyroelectric contribution can enhance the transient current response under modulated illumination [35, 36]. Previous studies have improved photovoltaic–pyroelectric photodetection using additional functional layers, nanostructured light‐trapping designs, or modified junction architectures [37, 38, 39]. Although these approaches can enhance charge transport, optical absorption, or interfacial separation, they may also increase fabrication complexity or reduce optical transparency, particularly when opaque or highly absorbing components are introduced [39, 40, 41, 42]. Therefore, an external and reversible modulation strategy that enhances device performance without changing the original solid‐state heterojunction is desirable for transparent self‐powered photodetectors.

Water offers a simple and physically meaningful medium for light‐reactive modulation. When water contacts the device surface, it can influence the photodetector through two distinct effects. First, because the refractive index of water is higher than that of air, water can reduce the refractive‐index contrast at the surface and suppress Fresnel reflection. This improves optical coupling into the device and increases the transmitted light intensity. Second, at the oxide semiconductor/water interface, surface charges and mobile ions in water can form an electric double layer (EDL) [30, 43, 44, 45]. This EDL does not directly increase optical transparency, but it can modify the surface potential and local electric field near the ZnO/water interface. Such electrostatic modulation can influence band bending, carrier separation, interfacial recombination, and transient polarization‐related charge transport [46, 47].

Here, we investigate the effect of direct water contact on the photovoltaic–pyroelectric response of the transparent FTO/ZnO/NiO/AgNW/ZnO photodetector. Compared with operation in air, the water contact improves optical transmission through reduced surface reflection and enhances the self‐powered photoresponse through interfacial electrostatic modulation. The device exhibits increased photocurrent, improved responsivity and detectivity, and stronger transient pyroelectric peaks under pulsed illumination. These results indicate that water acts not only as an operating environment but also as an external optical and electrostatic modulation medium. Compared with approaches that use additional functional layers or modified junction structures, the present approach uses the surrounding water interface to improve optical coupling and modify the interfacial electrostatic environment without changing the original heterojunction structure. This approach provides a simple external modulation strategy for enhancing the response of transparent self‐powered photodetectors. Supplementary Video S1 and Video S2 provide visual demonstrations of water‐compatible operation, including self‐powered capacitor charging, LED operation, and real‐time underwater photodetection. This work provides insight into water‐coupled interfacial modulation and suggests a promising strategy for transparent self‐powered sensing, smart windows, and submerged optoelectronic platforms.

## 2 Results and Discussion

### 2.1 Optical Properties of TPV

Figure 1a illustrates a schematic of the transparent photovoltaic (TPV) photodetector with an FTO/n‐ZnO/p‐NiO/AgNW/ZnO structure. In this device configuration, incident light enters through the FTO glass substrate. Owing to the wide‐bandgap nature of the FTO, ZnO, and NiO layers, high‐energy ultraviolet (UV) photons of wavelength (λ < 380 nm) are primarily absorbed, whereas visible light in the 380–780 nm range is transmitted through the oxide‐based layered heterojunction device. This wavelength‐selective optical behavior enables the device to maintain visible transparency while utilizing UV light for photoresponse. The AgNW network was used as a transparent conductive electrode (TCE) to improve electrical conduction while minimizing optical loss [48, 49]. In addition, the top ZnO layer was deposited on the AgNW network to protect the nanowires from oxidation and to improve the structural stability of the TCE [50, 51]. As a result, the device can simultaneously provide visible‐light transparency, ultraviolet absorption, and photovoltaic charge generation.

**FIGURE 1:**
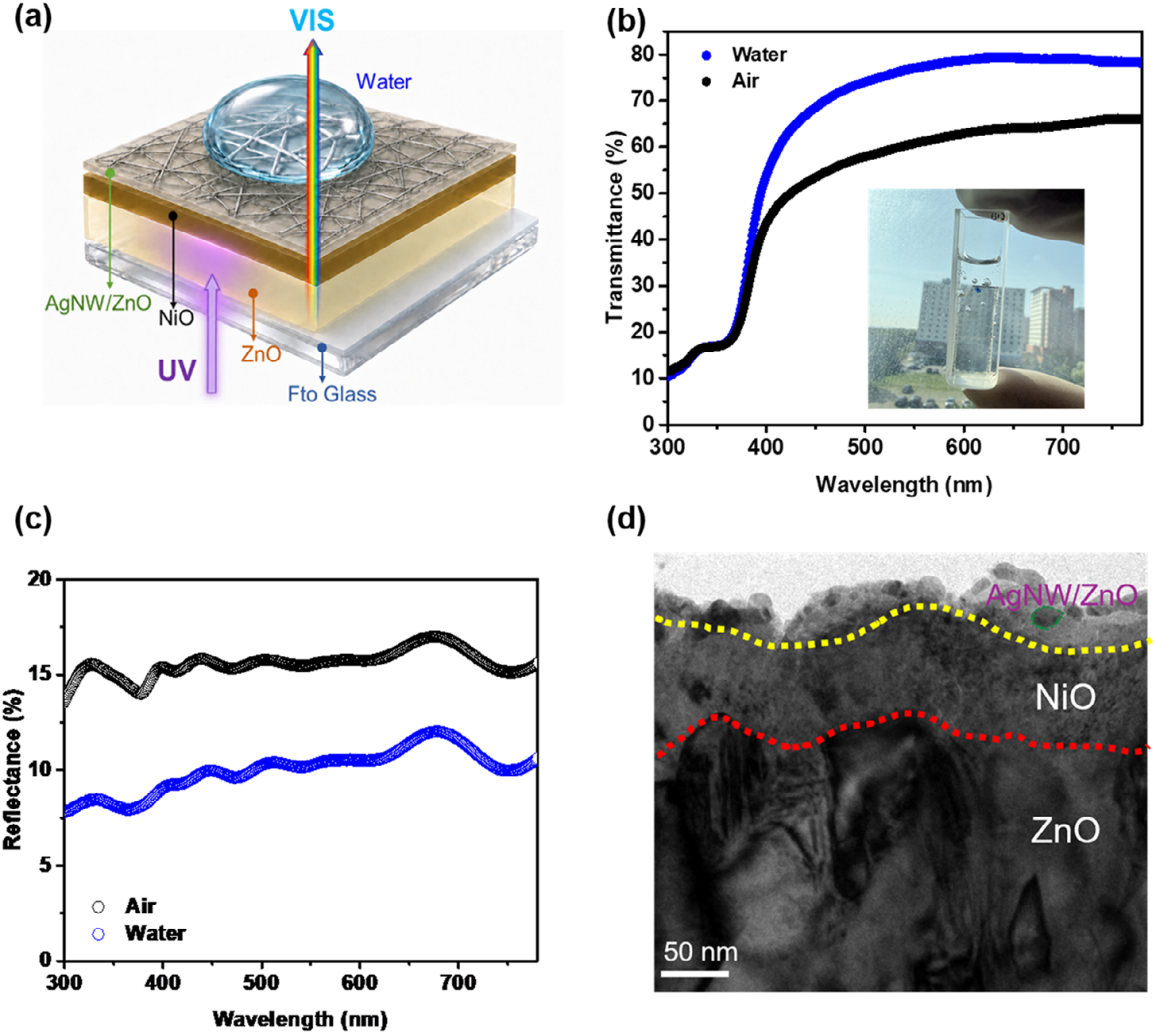
(a) Schematic illustration of the water‐assisted transparent self‐powered photodetector with an FTO/ZnO/NiO/AgNW/ZnO architecture, showing UV illumination through the FTO side and water contact at the top ZnO surface. (b) Optical transmittance spectra of the device measured in air and under water contact conditions. (c) Optical reflectance spectra in air and under water contact conditions, showing reduced surface reflection due to refractive‐index matching at the water/device interface. (d) Cross‐sectional TEM image of the FTO/ZnO/NiO/AgNW/ZnO multilayer structure, showing the AgNW‐based upper electrode with an ultrathin ZnO protective layer. The schematic illustration in (a) was generated using Google Gemini and subsequently edited by the authors.

Figure 1b compares the optical transmittance spectra of the device under air and water contact conditions. Both conditions exhibit low transmittance in the ultraviolet region, which is attributed to absorption by the FTO/ZnO/NiO structure. As the wavelength increases into the visible region, the transmittance gradually increases, confirming the transparent characteristics of the device [52]. The average visible transmittance (AVT) was calculated over the wavelength range of 380–780 nm using the following equation:

(1) $$AVT=\frac{\int_{380\;nm}^{780\;nm}T\left(\lambda \right)\;P\left(\lambda \right)\;S\left(\lambda \right)d\lambda }{\int_{380\;nm}^{780\;nm}P\left(\lambda \right)\;S\left(\lambda \right)\;d\lambda }\;$$

where *T(λ)* is the measured transmittance of the device, *P(λ)* is the photopic response of the human eye, and *S(λ)* is the AM 1.5G solar spectrum. The AVT (%) values of the device were calculated to be 61.0% under air and 77.02% under water, corresponding to an increase of approximately 16% in the visible region. The enhanced transmittance under water contact can be attributed to the reduction of optical reflection from the device. As shown in Figure 1c, the overall reflectance of the TPV photodetector decreases under water contact conditions compared with the air exposed state, indicating that less incident light is reflected from the device. This reduced reflection allows more light to enter and pass through the transparent heterojunction structure, leading to the improved transmittance under water contact.

To further examine the optical basis of the reduced reflection, the wavelength‐dependent effective refractive index of a 10 nm‐thick ZnO film deposited under the same conditions as the top ZnO layer was characterized by spectroscopic ellipsometry, as shown in Figure S2.

In the air exposed state, incident light enters the device through an air/ZnO interface, whereas under water contact, this interface is replaced by a water/ZnO interface. According to the Fresnel relation, the interfacial reflectance is governed by the difference between the refractive indices of the incident medium and the top surface material. Because the refractive index of water is higher than that of air, the difference between the refractive indices of the incident medium and ZnO becomes smaller when the surface is covered with water [47]. Therefore, the Fresnel reflection at the top ZnO interface is suppressed under water contact. The detailed Fresnel relation and quantitative estimation for this top‐interface reflection are provided in the Supplementary Note associated with Figure S2. This result indicates that water contact improves optical coupling into the TPV heterostructure by reducing surface reflection.

At the photoexcitation wavelength of 365 nm, the measured reflectance decreased from 14.409% in air to 7.939% under water contact, while the transmittance changed from 18.511% to 18.999%. Using the energy‐conservation relation *A*  =  1 − *R* − *T*, the corresponding total optical absorption increased from approximately 67.08% to 73.06%, corresponding to an approximately 8.9% increase at 365 nm. The corresponding transmittance, reflectance, and absorptance values at 365 nm are summarized in Figure S3. This wavelength‐specific optical enhancement is more directly relevant to the UV photoresponse than the visible‐range AVT increase.

The multilayer structure was examined by cross‐sectional TEM (Figure 1d), and the ZnO‐coated AgNW top electrode by top‐view scanning electron microscopy (Figure S4). As shown in Figure 1d, the FTO/n‐ZnO/p‐NiO/AgNW/ZnO device exhibits a well‐defined stacked architecture on the FTO glass substrate. The n‐ZnO and p‐NiO layers have thicknesses of approximately 100 and 30 nm, respectively, forming the main transparent p–n heterojunction responsible for self‐powered photodetection. The continuous ZnO/NiO interface indicates the formation of a compact oxide heterojunction, while the AgNW/ZnO upper layer serves as a transparent current‐collecting electrode. The AgNW upper electrode is covered by an ultrathin ZnO protective layer, which was introduced to protect the nanowire network and stabilize the transparent electrode. The nonuniform morphology of the upper region reflects the networked AgNW structure and the ultrathin ZnO capping layer.

### 2.2 Water‐Assisted Photovoltaic Properties

The vast aquatic environment covering nearly 71% of the Earth's surface represents an underexplored frontier for solar energy harvesting. Realizing efficient photovoltaic operation under direct water contact requires a fundamental understanding of how the semiconductor–water interface influences charge separation, carrier transport, and interfacial electric fields. To address this challenge, the water‐assisted photovoltaic characteristics of the transparent photovoltaic (TPV) device were systematically investigated, revealing the critical role of water in modulating its self‐powered output performance.

Figure 2 presents the proposed operating mechanism at the semiconductor–water interface and water‐assisted photovoltaic characteristics of the transparent ZnO/NiO heterojunction. As illustrated in Figure 2a, the FTO/ZnO/NiO/AgNW/ZnO heterostructure forms a transparent p–n junction, where *ϕ*, *χ*, and *E_g_* represent the work function, electron affinity, and bandgap energy, respectively [22, 53]. Owing to the wide bandgaps of ZnO (∼3.25 eV) and NiO (∼3.6–4.0 eV), the device remains highly transparent in the visible region while efficiently harvesting UV light. Upon UV illumination, photogenerated electron–hole pairs are primarily generated within the ZnO layer and subsequently separated by the built‐in electric field across the ZnO/NiO heterojunction. Consequently, electrons are transported toward the FTO electrode through the n‐type ZnO layer, whereas holes are extracted through the p‐type NiO layer toward the AgNW/ZnO transparent electrode, resulting in a photovoltaic current in the circuit without an external bias.

**FIGURE 2:**
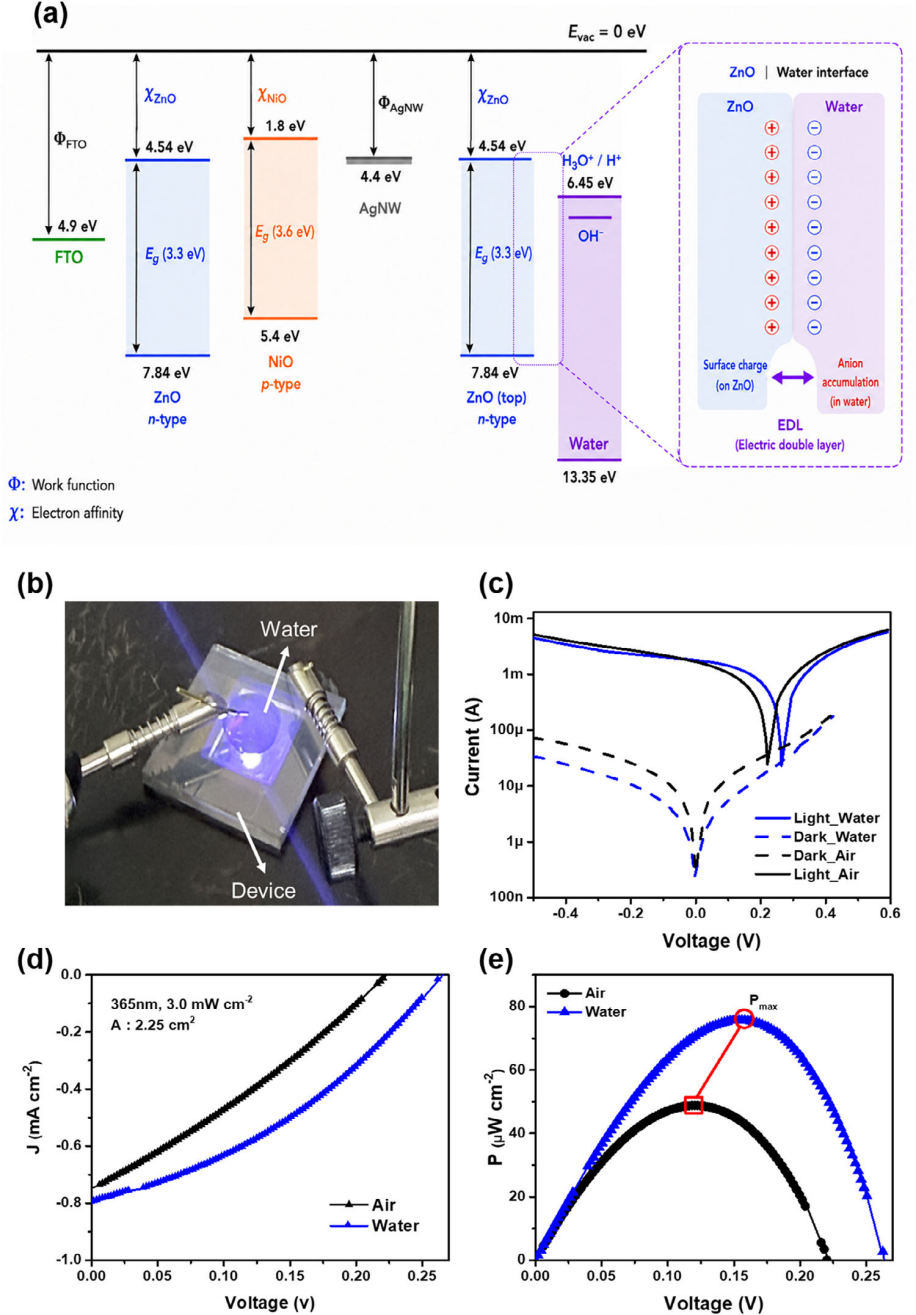
(a) Schematic energy‐level diagram of the FTO/ZnO/NiO/AgNW/ZnO device and the water contact interface, illustrating the relative work functions and band‐edge positions of the constituent layers. The top ZnO layer is directly exposed to water. (b) Photograph of the photovoltaic measurement configuration under UV illumination with water placed on the active device surface. (c) *I–V* characteristics plotted on a logarithmic current scale under dark and 365 nm UV‐illuminated conditions in air and under water contact. (d) *J–V* curves and (e) *P–V* curves of the TPV device under 365 nm UV illumination in air and under water contact. The UV light intensity and active area were 3.0 mW cm^−^^2^ and 2.25 cm^2^, respectively.

Unlike conventional photovoltaic operation in air, when water contacts the top ZnO surface, mobile cations and anions in the interfacial water region redistribute near the ZnO/water interface, leading to the formation of an electric double layer (EDL) [44, 45, 53, 54]. The resulting EDL‐induced interfacial polarization modifies the surface potential, charge distribution, and local band bending of ZnO. This additional electrostatic modulation complements the built‐in electric field of the ZnO/NiO heterojunction by facilitating carrier separation and extraction while suppressing interfacial recombination and leakage pathways. Consequently, the combined effects of the intrinsic p–n junction field and the water‐induced interfacial field contribute to the enhanced self‐powered photovoltaic response under water contact conditions [35, 47, 53, 54].

Figure 2b presents the TPV measurement configuration under water contact. The bottom FTO electrode was connected to the negative probe, while the top AgNW/ZnO electrode was connected to the positive probe. The photovoltaic characteristics were measured under both air exposed and water contact conditions using the same device configuration to directly compare the effect of water on the self‐powered output of the device.

Figure 2c shows the *I–V* characteristics of the device measured under air and water contact conditions. At *λ* = 365 nm UV illumination, both devices exhibited clear photovoltaic behavior without external bias, confirming self‐powered operation. The open‐circuit voltage (*V_oc_*) increased from 0.223 V in air to 0.265 V under water contact. The short‐circuit current density (*J_sc_*) also increased from 0.751 to 0.797 mA cm^−2^, corresponding to an increase in short‐circuit current (*I_sc_*) from 1.69 to 1.79 mA for an active area of 2.25 cm^2^. These results indicate that water contact enhances both photovoltage generation and photocurrent output in the TPV device.

The increase in *V_oc_* can be understood using the photovoltaic diode relation [30, 55, 56],

(2) $$V_{oc}=\frac{n\;k_{B}\;T}{q}\;\ln\left(\frac{J_{sc}}{J_{d}}\right)$$

where n is the ideality factor, *k_B_* is the Boltzmann constant, *T* is the absolute temperature, q is the elementary charge, *J_sc_* is the short‐circuit current density, and *J_d_* is the dark current density. According to Equation (2), *V_oc_* increases with increasing *J_sc_* and decreasing dark‐current contribution. In this device, the dark current decreased from approximately 20 nA in air to 10 nA under water contact. When water contacts the top ZnO surface, mobile ions in the adjacent water redistribute near the oxide surface, forming an interfacial electric double layer (EDL) and altering the local charge environment at the ZnO/water interface. The reduced dark current, together with the increased shunt resistance from 173.0 to 311.9 Ω, indicates that leakage pathways are suppressed under water contact. Therefore, the enhanced *V_oc_* results from the simultaneous increase in photogenerated current and reduction in leakage current under the water contact condition.

According to Equation (2), the contributions of the increased *J_sc_* and reduced dark current to the *V_oc_* enhancement can be compared using the corresponding logarithmic terms. In this device, *J_sc_* increased from 0.751 to 0.797 mA cm^−2^, corresponding to a ratio of 1.061 and a logarithmic term of 0.0595. The dark current decreased from approximately 20 to 10 nA, corresponding to a ratio of 2 and a logarithmic term of 0.6931. Therefore, approximately 7.9% of the total logarithmic change is associated with the increase in *J_sc_*, whereas approximately 92.1% is associated with the reduction in dark current. This result indicates that the enhanced *V_oc_* is mainly related to the reduction in leakage current under water contact.

The improvement in photovoltaic output is further supported by the extracted resistance parameters. The series resistance (*R_s_*) decreased from 95.2 Ω in air to 79.0 Ω under water contact, indicating reduced transport resistance and improved carrier extraction. In contrast, the shunt resistance (*R_sh_*) increased from 173.0 to 311.9 Ω, suggesting suppressed leakage pathways across the device. In a photovoltaic device, a low *R_s_* reduces resistive loss, while a high *R_sh_* limits leakage current [56]. Therefore, the simultaneous decrease in *R_s_* and increase in *R_sh_* contributes to the improved fill factor (*FF*) and output power under water contact.

(3) $$FF=\left(\frac{V_{max}\times J_{max}}{V_{OC}\;\times J_{SC}}\right)\;\times 100\%$$

where *V_max_* and *J_max_* are the voltage and current density at the maximum power point, respectively. In air, *V_max_* and *J_max_* were 0.12 V and 0.405 mA cm^−2^, respectively. Under water contact, *V_max_* and *J_max_* increased to 0.156 V and 0.486 mA cm^−2^, respectively. As a result, the *FF* increased from 29.2% in air to 35.8% under water contact. This increase indicates that the water contact condition provides a more favorable output curve by reducing internal loss and suppressing leakage current [57].

Figure 2d shows the *J–V* characteristics under 365 nm UV illumination. Compared with the air exposed device, the water contact device exhibited an overall enhanced photovoltaic response, as evidenced by the increased photovoltage and photocurrent density. This improvement is important for self‐powered operation because the photovoltaic voltage and current serve as the internal driving output without an external power supply [58]. Figure 2e presents the corresponding *P–V* curves. The maximum output power density (*P_max_*) increased from 48.8 µW cm^−2^ in air to 75.8 µW cm^−2^ under water contact. In addition, the maximum power point shifted from 0.120 V in air to 0.156 V under water contact, while *J_max_* increased from 0.405 to 0.486 mA cm^−2^. These results indicate that water contact improves not only the short‐circuit output but also the actual power‐generating region of the TPV device.

The power conversion efficiency under UV illumination was calculated using the equation.

(4) $$PCE=\left(\frac{J_{sc}\times V_{oc}\times FF}{P_{hv}}\right)\;\times 100$$

where *P_hv_* is the incident UV light intensity. In this measurement, the irradiation intensity was 30 W m^−2^. Under identical device area and illumination conditions, the *PCE* increased from 1.63% in air to 2.53% under water contact. This increase is attributed to the simultaneous enhancement of *V_oc_*, *J_sc_*, and *FF*, which results from improved photovoltage generation, more efficient carrier extraction, and reduced leakage current under water contact. Therefore, water contact effectively improves the self‐powered photovoltaic performance of the TPV device. The detailed role of water‐induced EDL formation in the performance enhancement will be discussed in a later section. The photovoltaic parameters extracted under air and water contact conditions are summarized in Table 1.

**TABLE 1:** Photovoltaic parameters of the TPV device under air and water contact conditions.

condition	*V_oc_* (V)	*I_sc_* (mA)	*J_sc_ (*mA cm^−2^)	*V_max_* (V)	*J_max_* (mA cm^−2^)	*P_max_* (µW cm^−2^)	*R_s_* (Ω)	*R_sh_* (Ω)	*FF* (%)	*PCE* (%)
Air	0.223	1.69	0.751	0.12	0.405	48.8	95.2	173	29.2	1.63
Water	0.265	1.79	0.797	0.156	0.486	75.8	79	311.9	35.8	2.53

### 2.3 Water‐Enhanced Pyroelectric Photoresponse

The transient photoresponse of the TPV device was investigated under pulsed UV illumination to evaluate the pyroelectric contribution. The measurements were performed using a 365 nm UV source at a pulse frequency of 0.5 Hz. The UV light intensity was controlled at 0.6, 0.9, and 1.2 mW cm^−2^, and identical illumination conditions were applied to both the air and water contact states. The underlying mechanism of pyroelectric current generation under periodic light‐on/off cycles is schematically illustrated in Figure S1. Light illumination is accompanied by thermal energy, resulting in heating during the light‐on state and cooling during the light‐off state. The absorbed thermal energy propagates from the illuminated side across the device thickness, generating a transient temperature change and temperature gradient. Because the electric polarization of ZnO is temperature‐dependent, this thermal variation induces a transient change in polarization and the associated bound charge, thereby producing sharp current peaks in the *I–t* response [30].

Figure 3a shows the *I–t* response of the device under air conditions. As the UV intensity increased, the steady‐state photocurrent increased due to the higher density of photo‐generated carriers produced under stronger illumination. The transient pyroelectric current peaks also increased when the UV intensity increased from 0.6 to 0.9 mW cm^−2^. However, when the intensity was further increased to 1.2 mW cm^−2^, the pyroelectric peak decreased compared with that at 0.9 mW cm^−2^, even though the photocurrent continued to increase. This indicates that, under air conditions, the photocurrent and pyroelectric current do not necessarily follow the same intensity‐dependent trend.

**FIGURE 3:**
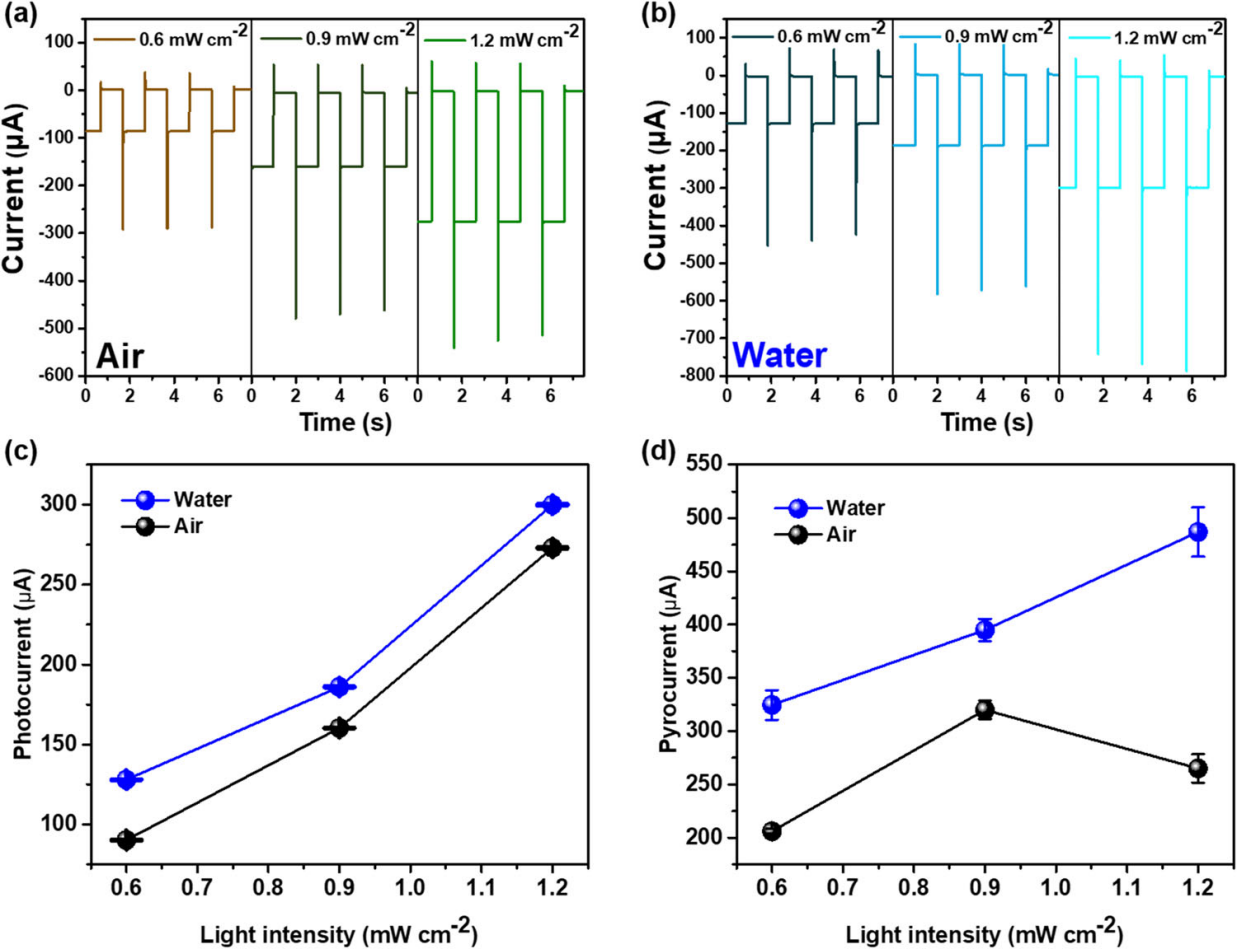
(a) *I–t* response of the device measured under air condition with 365 nm pulsed UV illumination at light intensities of 0.6, 0.9, and 1.2 mW cm^−^^2^. (b) *I–t* response of the device measured under water contact condition under the same pulsed UV illumination conditions. (c) Extracted photocurrent values under air and water contact conditions as a function of UV light intensity. (d) Extracted pyroelectric current under air and water contact conditions. The water contact device exhibited enhanced photocurrent over the measured intensity range, while the pyroelectric current increased continuously with UV intensity, unlike the air exposed device. Error bars in (c) and (d) represent the cycle‐to‐cycle standard deviation obtained from repeated transient pulses under identical measurement conditions; the error bars in (c) are smaller than the symbol size.

Figure 3b shows the *I–t* response under water contact conditions. Similar to the air condition, the photocurrent increased with increasing UV intensity. However, under water contact, both the photocurrent and pyroelectric current responses were more pronounced under identical illumination conditions. The enhanced photocurrent under water contact is consistent with the photovoltaic characteristics discussed in Section 2.2, where the reduced dark current, decreased *R_s_*, and increased *R_sh_* indicated suppressed leakage pathways and improved carrier extraction. Therefore, the larger photocurrent observed in the pulsed *I–t* response can be understood as a dynamic manifestation of the improved self‐powered TPV output under water contact. In addition, the pyroelectric response under water contact showed a continuous increase with UV intensity, suggesting that water contact provides an additional interfacial contribution to the transient current response beyond light‐induced photocarrier generation.

Figure 3c summarizes the extracted photocurrent values at 0.6, 0.9, and 1.2 mW cm^−2^. Under water contact, the photocurrent increased from 128 to 186 and 300 µA with increasing UV intensity. In comparison, the device under air conditions showed photocurrent values of 90, 160, and 274 µA, respectively. These values show that the water contact device exhibits higher photocurrent than the air condition device at all measured UV intensities.

Figure 3d compares the extracted pyroelectric current peaks, obtained by subtracting the steady‐state photocurrent level from the transient peak current. Figure 3d compares the transient current component obtained from the difference between the transient peak and the steady state photocurrent level. To further verify its pyroelectric origin, additional modulation frequency, excitation wavelength, and ZnO control measurements were performed, as shown in Figures S5, S6, and S7. The frequency and wavelength dependent responses, together with the selective appearance of transient peaks in the ZnO containing control device, support the assignment of the dominant transient component to the pyroelectric polarization of ZnO. Under water contact, the pyroelectric current increased from 324.6 to 395 and 487 µA as the UV intensity increased from 0.6 to 0.9 and 1.2 mW cm^−2^, respectively. In contrast, the device under air conditions showed pyroelectric current values of 206, 320, and 265 µA under the same illumination intensities. While the air condition device exhibited a maximum pyroelectric current at 0.9 mW cm^−2^ followed by a decrease at 1.2 mW cm^−2^, the water contact device maintained a continuous increase with increasing UV intensity. This comparison shows that water contact enhances the magnitude and intensity‐dependent evolution of the transient pyroelectric response during repeated light‐on/off cycles.

Overall, water contact improves both the photocurrent and pyroelectric current responses of the TPV device under pulsed UV illumination. This enhancement is consistent with an additional contribution from water‐induced EDL‐assisted interfacial modulation, and its contribution will be further evaluated using the effective pyroelectric coefficient, *P_i_*, in a later section.

### 2.4 Water‐Induced Interfacial Charge Modulation Mechanism

To clarify the origin of the water‐enhanced photoresponse, the interfacial charge behavior of the FTO/ZnO/NiO/AgNW/ZnO heterostructure under air and water contact conditions is schematically described [44, 47, 59]. Figure 4a illustrates the carrier separation process under air conditions. In the absence of water, the photoresponse is mainly governed by the built‐in electric field formed at the ZnO/NiO p–n heterojunction. Under UV illumination, photo‐generated electron–hole pairs are separated by this internal field; electrons are transported through the n‐type ZnO layer, while holes are transported through the p‐type NiO layer. Therefore, under the air condition, carrier separation and collection are primarily driven by the intrinsic p–n junction field of the ZnO/NiO heterostructure.

**FIGURE 4:**
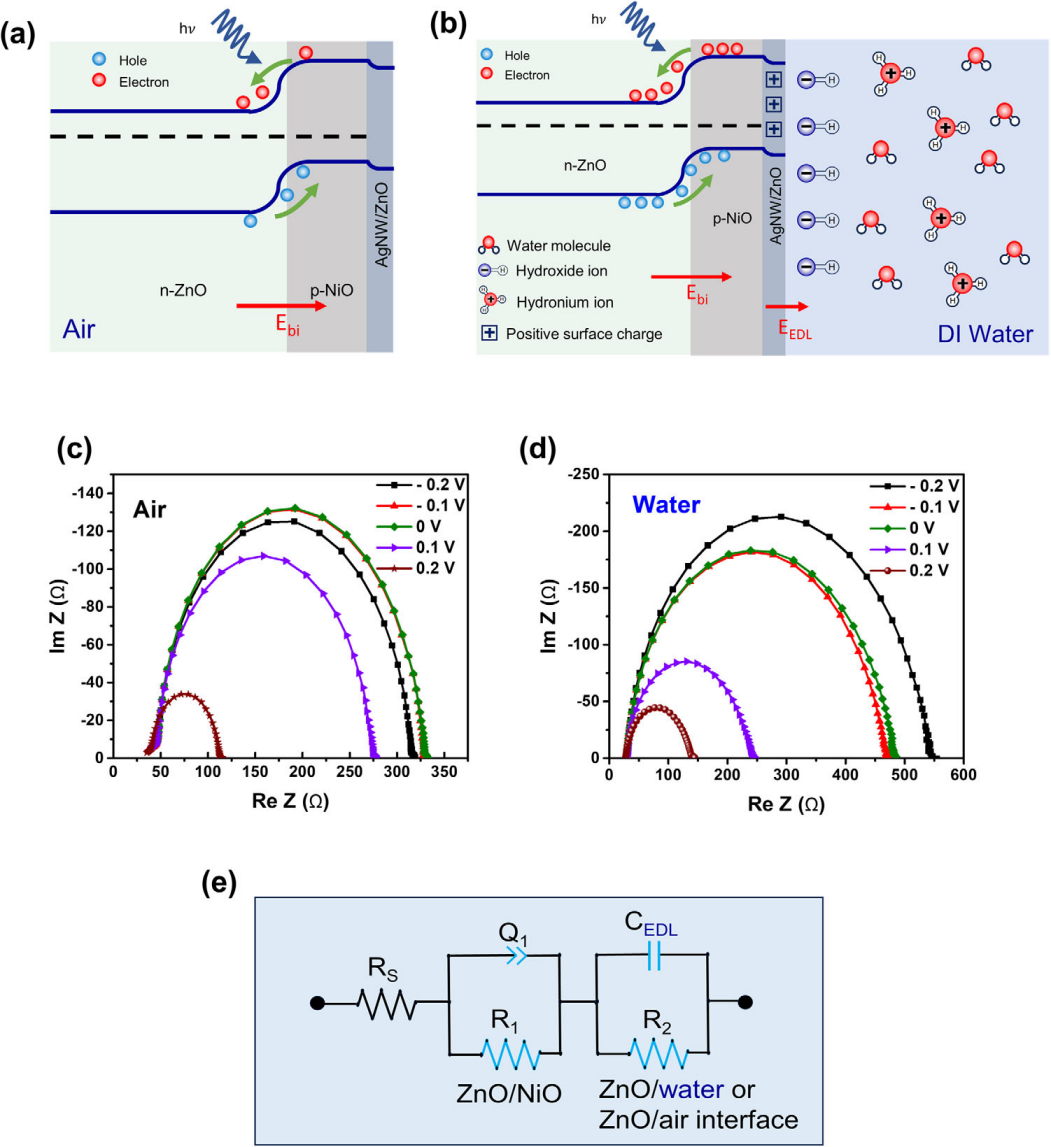
(a) Schematic energy‐band diagram illustrating carrier separation in the FTO/ZnO/NiO/AgNW/ZnO heterostructure under air exposed condition. (b) Schematic illustration of EDL formation at the ZnO/water interface and the resulting modulation of interfacial charge distribution under water contact. (c) Nyquist plots measured under dark conditions in air at different applied biases. (d) Nyquist plots measured under dark, water contact conditions at different applied biases, showing larger semicircular arcs and a left‐shifted high‐frequency intercept compared with the air exposed condition. (e) Equivalent‐circuit model used for fitting the EIS spectra, where *Rs* represents the series resistance, the *Q_1_*/*R_1_* branch corresponds to the ZnO/NiO junction‐related response, and the *C_EDL_/R_2_* branch represents the ZnO/external‐interface response under air or water contact.

Figure 4b shows the interfacial charge modulation under water contact conditions. When water contacts the top ZnO surface, mobile ions in water rearrange near the ZnO/water interface, leading to the formation of an electric double layer [43, 44, 45, 53]. This EDL induces interfacial charge accumulation and modifies the local surface potential of ZnO. Unlike the air condition, the water contact interface introduces an additional local electric field at the ZnO surface through EDL formation. This EDL‐induced field can further assist the separation and collection of photo‐generated carriers, in addition to the built‐in field of the ZnO/NiO junction. As a result, carrier recombination and leakage loss are reduced, which is consistent with the decreased dark current and enhanced photovoltaic output observed under water contact.

The influence of this water‐induced interfacial modulation can be understood from the electric displacement relation given by Equation (5) [47, 60],

(5) $$D=\varepsilon_{0}E+P$$

where *D* is the electric displacement, *ε_0_* is the vacuum permittivity, *E* is the electric field, and *P* is the polarization. As per Equation (5), the electric displacement is governed by the electric‐field term and the polarization term. Therefore, when EDL formation at the ZnO/water interface strengthens the local electric field and interfacial polarization, the electric displacement can also be enhanced. This enhanced electric displacement supports more effective charge separation and reduces recombination/leakage‐related losses under water contact.

The connection between the enhanced electric displacement and the pyroelectric response can be described by the displacement current relation, as shown in Equation (6) [47, 60].

(6) Id=d/dt∫∫Dds

where *I_d_* is the displacement current, and *ds* is the surface element. Equation (6) indicates that displacement current is generated by the time‐dependent variation of electric displacement. Therefore, under pulsed UV illumination, EDL‐assisted enhancement of the local electric field and interfacial polarization can increase the time‐dependent variation of *D* during repeated light‐on/off cycles [33, 44]. This provides an electrical basis for the enhanced pyroelectric current observed under water contact.

To evaluate the interfacial electrical response under water contact, dark‐state electrochemical impedance spectroscopy (EIS) measurements of the TPV device were performed under air and water [39, 61]. Figure 4c and Figure 4d show the Nyquist plots measured in the dark at different applied biases under air and water, respectively. In both cases, the Nyquist spectra showed semicircular arcs whose size and shape varied with the applied bias, indicating that the impedance response includes both resistive and capacitive components in the heterostructure.

Compared with the air exposed device, the water contact device showed a larger impedance arc and a more pronounced imaginary impedance response over the measured bias range, while the high‐frequency intercept tended to decrease. These features suggest that the overall resistive–capacitive response of the device is modified under water contact, with a possible reduction in series resistance and an enhanced interfacial polarization contribution. However, because the raw Nyquist spectra reflect the combined responses of series transport, the ZnO/NiO junction, and the ZnO/external interface, each contribution cannot be separately identified from the raw spectra alone.

Therefore, the measured EIS spectra were further fitted using the equivalent‐circuit model shown in Figure 4e, and the extracted fitting parameters are summarized in Table 2 and Table 3. In this model, *R_s_* represents the series resistance associated with the device and contact pathways. The *Q_1_* ∥ *R_1_* branch describes the ZnO/NiO junction‐related response, where *Q_1_* is a constant‐phase element (CPE) accounting for non‐ideal capacitive behavior, *Q_1_*‐*Y* is its admittance coefficient, and *R_1_* is the corresponding junction‐related resistance. The *C_EDL_* ∥ *R_2_* branch represents the ZnO/external interface, where *C_EDL_* denotes the interfacial electric‐double‐layer capacitance, and *R_2_* represents the corresponding interfacial resistance under air or water contact.

**TABLE 2:** EIS parameters extracted from the air exposed device.

Bias (V)	*R_s_* (Ω)	*Q_1_‐Y*	*R_1_* (Ω)	*C_EDL_* (nF)	*R_2_* (Ω)	*χ^2^*	*R^2^*
−0.2	37.481	312.936 n	268.646	36.115	8.870	7.311992 × 10^−5^	0.999918
−0.1	37.386	309.009 n	281.591	36.492	8.893	9.001289 × 10^−6^	0.999975
0	37.486	310.074 n	282.883	36.672	8.859	1.099007 × 10^−5^	0.999972
0.1	37.199	318.281 n	228.336	38.126	8.493	6.224396 × 10^−5^	0.999865
0.2	34.268	492.857 n	72.980	57.122	5.375	1.149890 × 10^−5^	0.999971

**TABLE 3:** EIS parameters extracted from the water contact device.

Bias (V)	*R_s_* (Ω)	*Q_1_‐Y*	*R_1_* (Ω)	*C_EDL_* (nF)	*R_2_* (Ω)	*χ^2^*	*R^2^*
−0.2	29.364	1.873 µ	352.451	601.390	162.305	1.921256 × 10^−5^	0.9999976
−0.1	29.179	1.934 µ	300.591	605.327	138.603	4.877913 × 10^−5^	0.999859
0	29.254	622.873 n	396.845	581.691	142.483	8.826478 × 10^−6^	0.9999993
0.1	28.429	5.260 µ	123.565	488.056	91.223	1.822747 × 10^−5^	0.999947
0.2	27.558	13.278 µ	59.568	490.816	55.322	3.315546 × 10^−5^	0.999857

The corresponding reduced chi‐square (*χ^2^*) and coefficient of determination (*R^2^)* values are provided in the EIS fitting parameter tables.

Based on the fitting results, the water contact device exhibited a lower *R_s_* than the air exposed device, indicating reduced series loss along the device/contact/interfacial pathway. In addition, the *Q_1_‐Y* value increased under water contact, suggesting enhanced capacitive polarization near the p–n junction/interface region [46, 47, 62]. This result indicates that the electrical environment around the ZnO/NiO junction is modified under water contact, which is consistent with the enhanced photovoltaic response observed under UV illumination.

The interfacial charge‐storage behavior is further supported by the extracted *C_EDL_* values. As shown in Figure 4e, the *C_EDL_* values were much smaller for the air‐exposed device, whereas they increased significantly under water contact. Specifically, *C_EDL_* increased from approximately 36–57 nF in air to 488–605 nF under water contact. This large increase indicates that water contact strengthens the capacitive charge‐storage behavior at the ZnO/water interface, supporting EDL‐assisted interfacial charge accumulation.

To further distinguish the EDL‐assisted contribution from a simple physical water contact effect, additional ionic‐strength and pH‐controlled experiments were performed. The transient photovoltaic and pyroelectric responses increased systematically with NaCl concentration in the order Air < DI water < 0.1 M < 1 M < 2 M, while the fitted *C_EDL_* simultaneously increased from 0.582 µF for DI water to 2.552, 2.871, and 3.924 µF for 0.1, 1, and 2 M NaCl, respectively. These correlated changes indicate that the ZnO/liquid interfacial response is sensitive to the ionic environment rather than to physical water contact alone. The corresponding transient‐current responses and raw Nyquist spectra are presented in Figures S8 and S9, respectively, while the extracted EIS fitting parameters are summarized in Table S1 and Table S2.

The pH‐controlled measurements further showed that EDL‐related charge accumulation alone does not determine the device response. In the present measurement set, the pH 4 and pH 11 conditions exhibited distinct *C_EDL_*, *R_s_*, and transient‐response characteristics, suggesting that the device response is influenced not only by EDL‐related charge accumulation but also by the interfacial electrostatic and transport conditions. In addition, the apparent flat‐band potential *(V_FB_)* consistently followed *V*_FB,pH4_ < *V*_FB,DI_ < *V*_FB,pH11_, indicating a pH‐dependent modification of the coupled interfacial electrostatic state. The detailed pH‐dependent EIS fitting results and frequency‐dependent apparent *V_FB_* values are provided in Table S2 and Figure S10, respectively.

Consistent with the EIS fitting results, frequency‐dependent Mott–Schottky analysis further elucidates the water‐induced modification of the interfacial electrical environment, including changes in the effective carrier concentration and flat‐band potential (Figure S11). The water contact device exhibited a lower value of *1/C^2^* than that of the air exposed device, indicating an increase in the space‐charge capacitance of the heterojunction [63, 64, 65]. In addition, the effective carrier concentration increased under water contact, suggesting that the water interface enhances the charge response of the junction and provides a more favorable condition for carrier transport and collection [66]. Notably, the positive shift in flat‐band potential indicates a strengthened built‐in electric‐field environment, which can further promote photogenerated carrier separation under self‐powered operation [65, 67, 68].

Taken together, the original air/water comparison and the additional ionic‐strength and pH‐controlled experiments support an EDL‐assisted interfacial modulation mechanism. The ionic‐strength‐dependent increase in transient response and *C_EDL_* indicates that the interfacial response is not governed by physical water contact alone, while the contrasting pH‐dependent behavior further suggests that device performance depends not only on interfacial charge accumulation but also on the electrostatic and transport conditions established at the ZnO/liquid interface. These results provide additional experimental support for the active role of the ZnO/liquid interface in the water‐enhanced photovoltaic and pyroelectric responses, which is further discussed in Section 2.5.

### 2.5 Effective Pyroelectric Coefficient and Water‐Enhanced Transient Response

Based on the water‐induced interfacial charge modulation as discussed in Section 2.4, the effective pyroelectric coefficient was estimated to quantitatively evaluate the enhanced transient polarization response under water contact. As described by Equations (5) and (6), the transient current response is related to the time‐dependent variation of electric displacement, which is determined by the local electric field and polarization. Since water‐induced EDL formation modifies the local electric field and interfacial polarization at the ZnO/water interface, it can enhance the displacement‐current component generated during repeated light‐on/off cycles.

The pyroelectric coefficient represents the change in polarization with respect to temperature and is defined as Equation (7) [69, 70].

(7) $$P_{i}=\frac{dP}{dT}\;$$

where *P_i_* is the effective pyroelectric coefficient, *P* is the polarization, and *T* is the temperature. Under pulsed illumination, repeated heating and cooling induce temperature‐dependent polarization modulation. Therefore, a larger effective *P_i_* indicates stronger polarization modulation for a given temperature variation [30, 71].

The pyroelectric current can be described by Equation (8).

(8) $$I_{\mathrm{pyro}}=P_{i}\;A\cdot \frac{\mathrm{dT}}{\mathrm{dt}}$$

where *I_pyro_* is the pyroelectric current, *A* is the active area, and *dT/dt* is the temporal temperature variation. Under identical device geometry and pulsed illumination conditions, the larger pyroelectric current observed under water contact indicates an enhanced effective pyroelectric response [35, 72]. This enhancement is attributed to EDL‐assisted modulation of the local electric field and interfacial polarization at the ZnO/water interface. However, according to *I*_pyro_ = *P_i_* *A*(*dT*/*dt*), the transient current can also be affected by the temporal temperature variation and thermal boundary conditions. Therefore, the contribution of changes in *dT*/*dt* under direct water contact cannot be completely excluded.

Measurements were additionally conducted at 23, 50, and 70 °C under DI water contact using 365 nm illumination, with the corresponding thermographic images provided in Figure S12. Across these temperature conditions, the transient pyroelectric contribution varied noticeably, whereas only a relatively small change was observed in the photovoltaic component. The different temperature sensitivities of these two current components indicate that the transient signal is strongly associated with thermally induced polarization in the ZnO/NiO TPV device, consistent with a previous temperature dependent investigation of the same heterojunction system [30, 35]. At the same time, direct water contact modifies the thermal environment surrounding the device and may alter heat transfer across the interface. Such changes can influence *dT/dt* and consequently contribute to the measured pyroelectric current according to *I*_pyro_ = *P_i_* *A*(*dT*/*dt*). Therefore, the enhanced transient response under water contact should be interpreted by considering both the temperature dependent polarization response of the heterojunction and the altered thermal boundary condition. On this basis, the effective pyroelectric coefficient was further evaluated quantitatively over the selected temperature range.

For quantitative estimation, *P_i_* was calculated within a specific temperature range using Equation (9) [30, 69, 70].

(9) $$P_{i}=\int J\cdot \frac{dt}{dT}=\sigma /\mathrm{dT}$$

where *J* is the pyroelectric current density, *t* is time, *σ* is the charge density obtained by integrating *J* over time, and *dT* is the temperature interval. To accurately estimate *P_i_*, the steady photocurrent contribution was excluded from the total transient current, and only the pyroelectric current component was integrated.

Figure 5a–c shows the pyroelectric current responses measured at different temperatures under air conditions, while Figure 5d–f shows the corresponding responses under water contact conditions. The measurements were conducted at 23, 50, and 70 °C. Compared with the air condition, the water contact device exhibited larger transient pyroelectric current responses over the measured temperature range. The extracted current density was integrated over time to obtain *σ*, and *P_i_* was then estimated using Equation (9).

**FIGURE 5:**
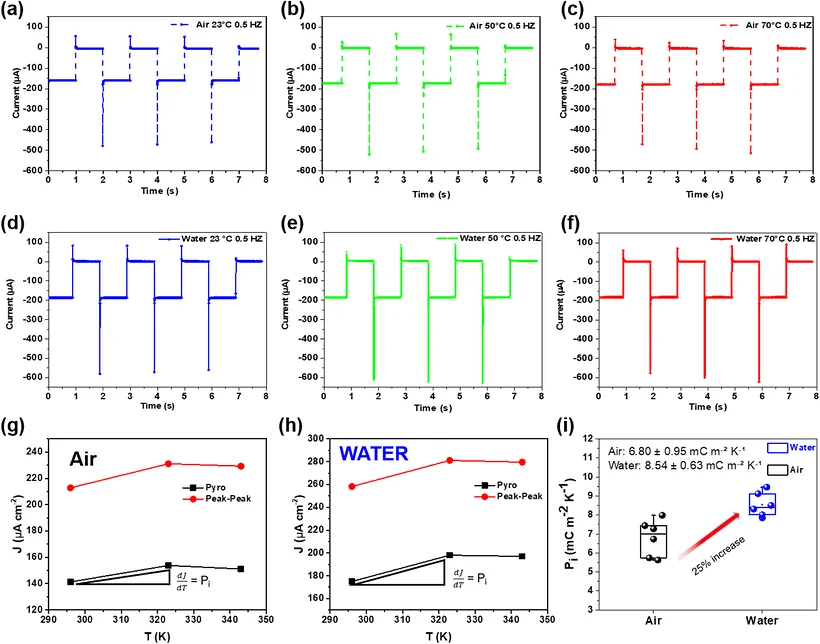
Pyroelectric current response measured under air condition at (a) 296 K, (b) 323 K, and (c) 343 K. (d) Pyroelectric current response measured under water contact condition at 296 K. (e) Pyroelectric current response measured under water contact condition at 323 K. (f) Pyroelectric current response measured under water contact condition at 343 K. (g) Temperature‐dependent pyroelectric current density and peak‐to‐peak current density extracted under air condition. (h) Temperature‐dependent pyroelectric current density and peak‐to‐peak current density extracted under water contact condition. (i) Comparison of the effective pyroelectric coefficient under air and water contact conditions, showing an approximately 25% increase under water contact. Data are presented as mean ± SD from six devices (n = 6).

Figure 5g,h summarize the temperature‐dependent pyroelectric current density and peak‐to‐peak current density under air and water contact conditions, respectively. Under air conditions, the pyroelectric current density changed from 141.3 to 153.8 and 151.1 µA cm^−2^ as the temperature increased from 296 to 323 and 343 K, respectively. The corresponding peak‐to‐peak current density changed from 212.8 to 231.1 and 229.3 µA cm^−2^. Under water contact, higher values were obtained over the entire temperature range, with pyroelectric current densities of 175, 198, and 197 µA cm^−2^ and peak‐to‐peak current densities of 258.2, 281.1, and 279.6 µA cm^−2^. These results show that water contact enhances the temperature‐dependent pyroelectric response, indicating stronger transient polarization modulation at the ZnO/water interface.

Figure 5i compares the estimated effective pyroelectric coefficients under air and water contact conditions. The reported room‐temperature pyroelectric coefficient of bulk ZnO is approximately 9.4 µC m^−2^ K^−1^, whereas the coefficient obtained from a ZnO/NiO heterojunction represents a device‐level effective value that additionally includes contributions from the junction built‐in electric field and heterojunction‐mediated thermal coupling [30]. In the present TPV device, the effective *P_i_* increased from approximately 6.8 ± 0.95 mC m^−2^ K^−1^ in air to 8.5 ± 0.63 mC m^−2^ K^−1^ under water contact (mean ± SD, n = 6), corresponding to an enhancement of approximately 25%. The increase in effective *P_i_* indicates a stronger device‐level transient polarization response under water contact. Although the instantaneous pyroelectric current depends directly on *dT/dt*, the extracted effective *P_i_* reflects the overall polarization response across the measured temperature range and therefore should not be explained solely by changes in the instantaneous thermal rate. Together with the interfacial charge modulation discussed in Section 2.4, these results suggest that EDL‐assisted modulation of the interfacial electric field and polarization contributes to the enhanced transient response. Accordingly, the water‐enhanced transient response is interpreted as reflecting contributions from both EDL‐assisted interfacial polarization modulation and modified thermal boundary conditions. This increase confirms that water contact enhances the effective polarization response of the TPV device. Overall, the *P_i_* analysis quantitatively supports the water‐enhanced transient pyroelectric response. The EDL formed at the ZnO/water interface modifies the local electric field and interfacial polarization, leading to a larger temperature‐dependent polarization change under pulsed illumination. As a result, both the effective pyroelectric coefficient and pyroelectric current are enhanced under water contact conditions.

### 2.6 Photodetection Performance and Response Dynamics

The photoresponse performance of the TPV‐based photodetector was evaluated under air and water contact conditions. The responsivity and specific detectivity were calculated using Equations (10) and (11), respectively [73].

(10) $$R\;=\frac{I_{Light}-I_{dark}}{A\cdot P}$$

(11) $$D^{*}=\frac{R\sqrt{A}}{\sqrt{\frac{4k_{B}T}{R_{sh}}+2qI_{dark}}}$$

where *R* is the responsivity, *I_light_* and *I_dark_* are the currents under illumination and dark conditions, respectively, *A* is the active area, *P* is the incident light intensity, *R_sh_* is the zero‐bias differential shunt resistance, *k_B_* is the Boltzmann constant, *T* is the absolute temperature, *q* is the elementary charge, and D* is the specific detectivity. Because the device operates under self‐powered conditions at 0 V, the detectivity was estimated by considering the thermal‐noise contribution associated with the shunt resistance together with the shot‐noise contribution. The zero‐bias differential shunt resistance, *R_sh_*, was extracted from linear fitting of the dark *I–V* characteristics in the near‐zero‐bias range from ‐0.02 to +0.02 V. The extracted *R_sh_* values were approximately 7.72 kΩ under air and 19.7 kΩ under water contact. Using these values together with the corresponding zero‐bias dark currents of approximately 20 nA and 10 nA, respectively, the specific detectivity was recalculated using an expression that accounts for both thermal noise and shot noise. Therefore, the responsivity represents the photocurrent generated per incident optical power, while the specific detectivity reflects the device capability to detect weak optical signals by accounting for both thermal noise and shot noise under zero‐bias operation.

Figure 6a compares the responsivity and specific detectivity of the device under air and water contact conditions. The responsivity increased from 76.5 mA W^−1^ in air to 93.1 mA W^−1^ under water contact, corresponding to an enhancement factor of approximately 1.217. At 365 nm, the total optical absorption increased from 67.08% to 73.06%, corresponding to an optical absorption enhancement factor of approximately 1.089 (73.06/67.08). As an approximate normalization for the measured optical absorption change, the responsivity enhancement factor was divided by the optical absorption enhancement factor, giving a remaining response enhancement factor of approximately 1.117 (1.217/1.089). This remaining enhancement indicates that the increase in responsivity cannot be explained by the optical absorption change alone and is consistent with an additional contribution from improved carrier separation and collection under water contact.

**FIGURE 6:**
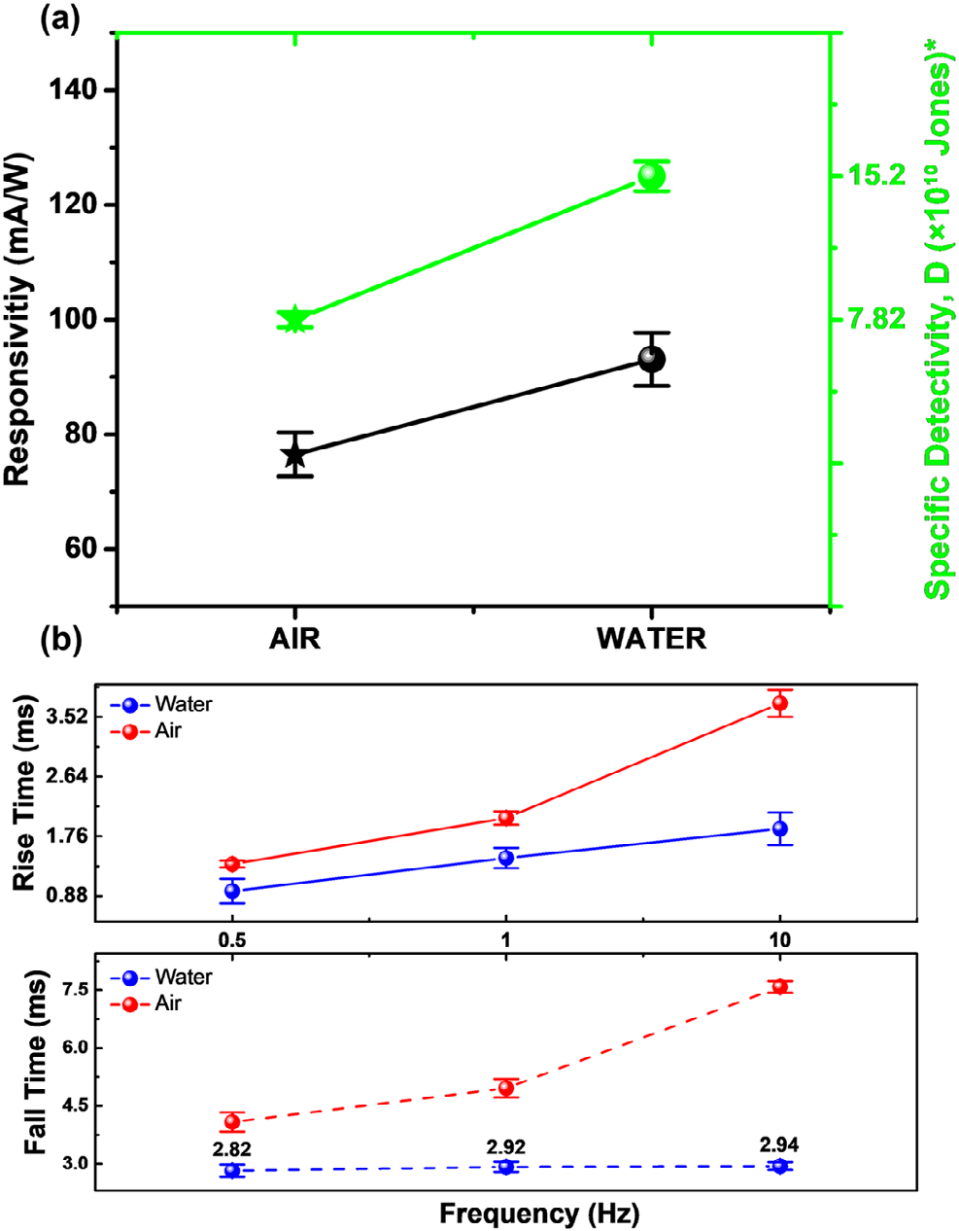
(a) Responsivity and specific detectivity of the device under air exposed and water contact conditions, calculated under 365 nm UV illumination with an optical intensity of 0.9 mW cm^−2^. Both parameters were enhanced under water contact, with the responsivity increasing from 76.5 to 93.1 mA W^−1^ and the specific detectivity increasing from 7.82 × 10^10^ to 1.52 × 10^11^ Jones. (b) Rise and fall times measured under 365 nm UV illumination at an optical modulation frequency of 0.5 Hz. The water contact device showed faster rise behavior while maintaining a nearly unchanged fall time, indicating a stable temporal response under repeated UV illumination. Error bars in (a) represent a conservative ±5% relative variation based on repeated measurements on the same device, while those in (b) represent the cycle‐to‐cycle variation obtained from repeated transient measurements.

The specific detectivity also increased from 7.82 × 10^10^ Jones in air to 1.52 × 10^11^ Jones under water contact. Repeated measurements showed variations of less than 5% in both responsivity and specific detectivity, indicating that the observed enhancement was consistently maintained. This enhancement reflects not only the increased responsivity but also the reduced thermal and shot noise contributions resulting from the increased zero bias differential shunt resistance and decreased dark current under water contact [47].

Under the air condition, the rise time increased from 1.35 to 2.03 and 3.72 ms as the frequency increased from 0.5 to 1 and 10 Hz, respectively. In comparison, the water contact device exhibited shorter rise times of 0.953, 1.44, and 1.87 ms at the same frequencies. This result indicates that water contact enables faster photocurrent build‐up under pulsed illumination, which can be attributed to improved charge separation and carrier extraction at the ZnO/water interface.

The fall time also showed a clear improvement under water contact. Under air conditions, the fall time increased from 4.08 to 4.96 and 7.58 ms as the frequency increased from 0.5 to 1 and 10 Hz, respectively. In contrast, the water contact device showed shorter fall times of 2.82, 2.92, and 2.94 ms over the same frequency range. The nearly unchanged fall time indicates that the device maintains rapid current recovery, suggesting that the response speed is preserved under repeated optical modulation. Together with the improved responsivity and detectivity, these results demonstrate that water contact enhances both the magnitude and temporal response characteristics of the self‐powered photoresponse.

To further evaluate the device performance, the key photodetection parameters of this work were compared with those of previously reported self‐powered photodetectors, as summarized in Table 4 [61, 75, 76, 77, 78, 79, 80, 81]. Despite being operated under water contact conditions, the proposed device exhibited competitive responsivity, detectivity, and response speed compared with other self‐powered photodetectors. Under water contact, the device simultaneously maintains an AVT of 77.02%, zero bias operation, a responsivity of 93.1 mA W^−1^, a specific detectivity of 1.52 × 10^11^ Jones, and fast rise and fall times of 0.953 and 2.82 ms, respectively. This combination of transparency, self‐powered operation, and fast photoresponse is particularly relevant to transparent sensing systems that require operation in aqueous environments. These characteristics demonstrate the practical potential of the device for underwater photodetection and transparent environmental sensing.

**TABLE 4:** Comparison of reported transparent/self‐powered photodetectors. Device structure, AVT, wavelength (*λ*), bias, responsivity (*R*), detectivity (D*), response times, and photo/pyroelectric output are summarized. NR denotes not reported.

Device	AVT (%)	*λ* (nm)	Bias	*R* (mA W^−1^)	D* (Jones)	Rise/fall time	*I_pyro_* + *I_photo_*	Refs.
FTO/ZnO/NiO/AgNW/ZnO with water contact	77%	365	0 V	93.1	1.52 × 10^11^	0.953 / 2.82 ms	258 µW cm^−^^2^	This work
MoS_2_ with water contact	Opaque	470	0 V	24.6	2.85 × 10^8^	0.741 / 2.83 ms	2 nA	[61]
MoS_2_/WSe_2_	Opaque	532	0 V	200	1.5 × 10^12^	46 / 54 µs	NR	[75]
AgNW/ Cu_2_O(NiO)/ZnO/FTO	51%	365	0 V	0.98	7.2 × 10^1^ ^2^	42.5 / 56 µs	150 µW cm^−^^2^	[17]
ITO/CoOx/HfO_x_/SnO_2_/ZnO/ITO	75%	340∼350	NR	80.1	2.55 ×10^1^ ^1^	100 / 110ms	NR	[76]
ZnO/CuI/ZnS	70∼80%	372	0 V	43.85	3.84 ×10^14^	305 / 261ms	NR	[77]
AZO/GaN/ITO	NR	365	0 V	1.18 AW^−^^1^	1.75 ×10^1^ ^2^	31 / 38ms	NR	[78]
ITO/SAM/WS_2_/graphene	67%	325	0 V	1.58	3.29 ×10^9^	<200 ms / <200 ms	NR	[79]
ITO/Ag‐Ag(O)/ZnO/NiO/AgNWs	62.1%	365	0V	0.8 AW^−^^1^	NR	80 / 130 µs	3 µAcm^−2^	[80]
Co_3_O_4_/WS_2_	NR	395	0V	41.4	7.3 × 10^11^	39 / 51 ms	<250 nA	[81]

NR denotes not reported.

## 3 Conclusion

In this work, we demonstrated a water‐assisted transparent self‐powered photodetector based on an FTO/ZnO/NiO/AgNW/ZnO heterojunction, in which water functions as an active interfacial engineering medium rather than merely the surrounding environment. Compared with operation in air, water contact significantly improves both the optical and optoelectronic characteristics of the transparent oxide platform. The average visible transmittance increases from 61.0% to 77.02% owing to reduced optical reflection at the ZnO/water interface. Simultaneously, the photovoltaic performance is markedly enhanced, with the open‐circuit voltage from 0.223 to 0.265 V (≈18.8%), the short‐circuit current density from 0.751 to 0.797 mA cm^−2^ (≈ 6.1%), the fill factor from 29.2% to 35.8% (≈ 22.6%), and the power conversion efficiency from 1.63% to 2.53% (≈ 55.2%). These improvements are accompanied by a lower series resistance, an increase in shunt resistance, and a 55.3% enhancement in maximum output power, indicating more efficient charge extraction and suppressed leakage losses under water contact conditions. Electrochemical impedance spectroscopy reveals enhanced interfacial capacitance arising from electric double layer formation at the ZnO/water interface. Additional ionic‐strength and pH‐controlled experiments provided further evidence of changes in the ZnO/liquid interfacial electrical environment. The monotonic increase in *C_EDL_* with increasing NaCl concentration, together with the distinct responses observed under different pH conditions, suggests that the device performance is influenced not only by interfacial charge accumulation, but also by the electrostatic and transport conditions at the ZnO/liquid interface. The resulting interfacial field modulation strengthens transient polarization under pulsed UV illumination, resulting in an approximately 25% increase in the effective pyroelectric coefficient, from 6.8 to 8.5 mC m^−2^ K^−1^. Consequently, compared with air operation, the device exhibits higher photocurrent, a 21.7% increase in responsivity from 76.5 to 93.1 mA W^−1^ and a 1.94‐fold enhancement in specific detectivity from 7.82 × 10^10^ to 1.52 × 10^11^ Jones, together with faster response dynamics. These enhancements reflect the combined effects of improved optical coupling, enhanced carrier separation and collection, and reduced electrical noise under water contact. Furthermore, the device successfully generates electrical output and charges a capacitor without external bias under direct water contact condition, demonstrating its capability for autonomous operation in aqueous environments. Future research can extend this interfacial engineering strategy by exploring alternative oxide or hybrid passivation layers, alongside spectral and bandgap engineering, to improve long‐term stability, operational reliability, and optoelectronic performance under diverse underwater illumination conditions.

Overall, these findings establish water‐interface engineering as a simple yet powerful strategy for enhancing optical coupling, interfacial electrostatics, and charge transport in transparent oxide heterojunctions, providing a promising route toward high‐performance transparent optoelectronic devices for underwater photodetection, environmental monitoring, smart windows, and next‐generation self‐powered sensing technologies.

## 4 Materials and Methods

### 4.1 Device Fabrication

Commercial FTO‐coated glass substrates (735159, Aldrich, sheet resistance: 7 Ω/□) were used as the transparent bottom electrodes. The FTO substrates were sequentially sonicated in acetone, methanol, and deionized water for 8 min each, followed by drying with high‐purity N_2_ gas (99.9%) under high‐pressure purging. The oxide layers were deposited using a magnetron sputtering system (Solarlight Ltd.) at room temperature. Before deposition, the chamber was evacuated to a base pressure of 10^−^^6^ Torr. The ZnO/NiO heterojunction was fabricated by sequential sputtering of n‐type ZnO and p‐type NiO layers. The ZnO layer was deposited for 25 min by radio frequency (RF) magnetron sputtering using a ZnO target (Dasom RMS, purity: 99.99%) at an RF power of 200 W under an Ar flow rate of 50 sccm. The NiO layer was deposited for 30 min by direct current (DC) reactive sputtering using a Ni target (iTASCO, purity: 99.99%) at a DC power of 55 W under Ar and O_2_ gas flow rates of 20 and 3 sccm, respectively. During sputtering, the sample holder was rotated at 4 rpm to improve thickness uniformity. AgNWs dispersed in isopropanol were purchased from Nanopyrix (South Korea) and used as received. The AgNW dispersion, with an average nanowire diameter of approximately 20 nm and length of approximately 20 µm, was dropped onto the ZnO/NiO heterojunction and spin‐coated once at 2500 rpm to form a transparent conducting AgNW network. The effective thickness of the AgNW network was approximately 20–40 nm. Finally, a 10 nm ZnO layer was deposited on the AgNW network to protect the nanowires and stabilize the transparent top electrode. The final device structure was FTO/ZnO/NiO/AgNW/ZnO, with a device area of 1.5 × 1.5 cm^2^.

For the control experiments, FTO/ZnO/AgNWs and FTO/NiO/AgNWs devices were fabricated using the same corresponding ZnO, NiO, and AgNW processing conditions as those used for the main TPV device.

### 4.2 Characterization

The cross‐sectional morphology and elemental distribution of the fabricated device were analyzed using field‐emission transmission electron microscopy (FETEM, JEM‐2100F; JEOL). Cross‐sectional specimens were prepared using a focused ion beam system. The FETEM images were analyzed using GMS 3 digital microscopy software to confirm the multilayer structure of the FTO/ZnO/NiO/AgNW/ZnO device.

The optical transmittance spectra were measured using a UV–vis–NIR spectrophotometer (Shimadzu, UV‐2600) over the wavelength range of 300–1400 nm. For water contact optical measurements, the fabricated device was immersed in a water‐containing cuvette, and the transmittance was measured under the same optical configuration.

The wavelength‐dependent effective refractive index was obtained by spectroscopic ellipsometry of a 10 nm‐thick ZnO film deposited on an n‐Si substrate under the same deposition conditions used for the top ZnO layer of the device. The ellipsometric data were fitted using a Cauchy dispersion model in the transparent visible region, where the extinction coefficient *k* was assumed to be negligible.

For the additional ionic‐strength control experiments, NaCl solutions with concentrations of 0.1, 1, and 2 M were prepared using DI water. For the pH‐controlled measurements, acidic and alkaline aqueous solutions were prepared using DI water, HNO_3_, and KOH, with their relative amounts adjusted to obtain final pH values of 4 and 11, respectively. Untreated DI water was used as the approximately neutral reference.

The photovoltaic and electrical characteristics of the device were measured using a potentiostat/galvanostat (PGStat ZIVE SP2, WonA Tech). During the photovoltaic *I–V* measurements, the FTO bottom electrode was connected to the negative probe, while the AgNW/ZnO top electrode was connected to the positive probe. A 365 nm LED source (WLS‐LED‐0365–04) was used for UV illumination, and the incident light was adjusted to enter the device normal to the surface. The incident light intensity for photovoltaic *I–V* measurements was checked using a solar power meter (TES‐1333) and fixed at 3.0 mW cm^−2^. The open‐circuit voltage, short‐circuit current density, fill factor, series resistance, shunt resistance, maximum output power density, and power conversion efficiency were extracted from the measured *I–V* curves. For water contact measurements, two drops of deionized water were placed on the top ZnO surface using a syringe before measurement.

All aqueous measurements, including DI water, NaCl solutions, and pH controlled solutions, were performed at room temperature. Before each measurement, the aqueous solution was allowed to equilibrate with the ambient laboratory temperature, and the temperature was monitored using an Arduino UNO based temperature monitoring system and confirmed using a K‐type thermocouple. Measurements were performed with the solution temperature maintained within 22.5‐23.5 °C (23.0 ± 0.5 °C).

The transient photoresponse was measured under modulated 365 nm UV illumination using the same LED source and normal‐incidence configuration. The light modulation was controlled using a function generator (MFG 3013A, MCH Instruments). Additional transient measurements were performed as a function of modulation frequency (10–100 Hz) and excitation wavelength (365, 410, 490, 530, and 630 nm), together with FTO/ZnO/AgNWs and FTO/NiO/AgNWs control measurements. For transient *I–t* measurements, the incident UV irradiance was calibrated using a UV light meter (UV‐340A, Lutron; detection range: 290–390 nm) and controlled in the range of 0.6–1.2 mW cm^−2^. The responsivity and specific detectivity were calculated from the transient photocurrent response measured under 365 nm UV illumination at an optical intensity of 0.9 mW cm^−2^. For the zero‐bias detectivity calculation, the differential shunt resistance, *R_sh_*, was extracted from linear fitting of the dark I–V characteristics in the near‐zero‐bias range from ‐0.02 to +0.02 V. The extracted *R_sh_* values were approximately 7.72 kΩ under air and 19.7 kΩ under water contact. The specific detectivity was then recalculated at 300 K using an expression that accounts for both thermal noise and shot noise, together with the corresponding zero‐bias dark currents of approximately 20 nA in air and 10 nA under water contact. The rise and fall times were extracted from the *I–t* responses measured at optical modulation frequencies of 0.5, 1, and 10 Hz. Dark‐state EIS measurements were performed to examine the interfacial electrical response of the device. For the original air and DI water contact comparison, Nyquist spectra were measured over an applied‐bias range from −0.2 to 0.2 V. For the additional ionic‐strength and pH‐controlled experiments, Nyquist spectra were measured from −0.2 to 0.1 V. To directly correlate the impedance characteristics with the self‐powered transient‐current response, the quantitative comparison of the fitted EIS parameters was performed at 0 V. The EIS spectra were analyzed using the ZMAN analysis tool, and equivalent‐circuit fitting was conducted to extract the resistance and capacitance‐related parameters, including *R_s_*, *Q_1_‐Y*, *R_1_*, *C_EDL_*, and *R_2_*. Mott–Schottky measurements were also carried out under dark conditions at different frequencies of 250, 100, 50, 25, 20, and 15 kHz. For the additional pH‐controlled comparison, the apparent *V_FB_* was evaluated over the analyzed frequency range of 10–32 kHz to compare the relative electrostatic shifts among pH 4, DI water, and pH 11 conditions. The corresponding *1/C^2^*–*V* plots were analyzed using the ZMAN analysis tool, and the effective carrier density and flat‐band potential were extracted from the linear fitting region of the Mott–Schottky plots. For effective pyroelectric coefficient measurements, the device temperature was varied from 23 to 70 °C using a hot plate, and the surface temperature was calibrated using an infrared thermal camera (Fluke Ti95). The effective pyroelectric coefficient was calculated from the transient pyroelectric current and the corresponding temperature variation measured under 365 nm UV illumination at an optical intensity of 0.9 mW cm^−2^. For the measurements reported in this study, the optical, photovoltaic, transient photoresponse, EIS, Mott–Schottky, and photodetection measurements, together with the measurements at different modulation frequencies and excitation wavelengths and the control measurements under different ionic strength and pH conditions, were each conducted using one representative device (n = 1) for the corresponding measurement set. For all comparison measurements, including different light intensities, air and water contact conditions, ionic strength and pH conditions, modulation frequencies, and excitation wavelengths, the same physical device was consistently used throughout the compared conditions within the corresponding measurement set. Repeated transient cycles and repeated electrical measurements obtained from the same physical device were treated as technical repeats rather than as independent devices. For the effective pyroelectric coefficient analysis in Figure 5i, six independently fabricated devices were evaluated (n = 6), and the data are presented as mean ± SD.

## Author Contributions

**Seunghee Cho**: writing – original draft, writing – review and editing, visualization, validation, methodology, software, formal analysis, data curation. **Vivek Mohan More**: writing – original draft, writing – review and editing, methodology, validation, formal analysis. **Sanh Vo Thi**: methodology, validation, visualization. **Md Arifur Rahman Barno**: methodology, validation, visualization. **Seojun Jeon**: methodology, validation, visualization, formal analysis, software, data curation. **Yebean Cho**: methodology, validation, visualization, software, formal analysis, data curation. **Joondong Kim**: conceptualization, writing – original draft, writing – review and editing, project administration, supervision, resources.

## Conflicts of Interest

The authors declare no conflicts of interest.

## Supporting information




**Supporting File 1**: advs78373‐sup‐0001‐SuppMat.docx.

**Supporting File 2**: advs78373‐sup‐0002‐VideoS1.mp4.

**Supporting File 3**: advs78373‐sup‐0003‐VideoS2.mp4.

## Data Availability

The data that support the findings of this study are available from the corresponding author upon reasonable request.
