# Supplementary material for: Water‐Coupled Interfacial Modulation Enhances Photovoltaic and Pyroelectric Responses in Transparent Self‐Powered ZnO/NiO Photodetectors
Source: Adv Sci (Weinh). 2026 Oct 22:e78373. Online ahead of print. doi: 10.1002/advs.78373 (PMC13690973; doi:10.1002/advs.78373)
Supplement: Supplementary file 1 [file ADVS-9999-e78373-s001.docx]

Supporting Information

**Water-Coupled Interfacial Modulation Enhances Photovoltaic and Pyroelectric Responses in Transparent Self-Powered ZnO/NiO Photodetectors**

*Seunghee Cho^1,2^, Vivek Mohan More^1,2^, Sanh Vo Thi^1,2^, Md Arifur Rahman Barno ^1,2^, Seojun Jeon^1,2^, Yebean Cho^1,2^ and Joondong Kim^1,2,^**

*^1^Photoelectric and Energy Device Application Lab (PEDAL), Multidisciplinary Core Institute for Future Energies (MCIFE), Incheon National University, 119 Academy Rd. Yeonsu, Incheon, 22012, Korea (Rep. of)*

*^2^Department of Electrical Engineering, Incheon National University, 119 Academy Rd. Yeonsu, Incheon, 22012, Korea (Rep. of)*

*Corresponding author: J. Kim ([joonkim@incheon.ac.kr](mailto:joonkim@incheon.ac.kr))

**Fig. S1**. Schematic illustration of photovoltaic and pyroelectric responses under steady and pulsed illumination. Under pulsed illumination, light-induced heating and cooling generate transient pyroelectric current in addition to the steady photocurrent.

**Supplementary Note: Electrical responses under steady and pulsed illumination**

Under steady illumination, the device mainly operates through the photovoltaic effect. Photogenerated electron–hole pairs are separated by the built-in electric field formed at the ZnO/NiO heterojunction, resulting in a steady photocurrent without an external bias. In contrast, under pulsed illumination, the repeated light ON/OFF process induces transient heating and cooling in the ZnO layer, allowing an additional pyroelectric response to be generated together with the photovoltaic current.

The origin of this pyroelectric response is the temperature-dependent spontaneous polarization of ZnO. Owing to its non-centrosymmetric wurtzite crystal structure, ZnO possesses spontaneous polarization, which induces asymmetric charge distribution inside the material and leads to the accumulation of opposite surface charges. Under equilibrium conditions, these polarization charges are balanced by compensation charges near the surface. When the temperature of ZnO changes under pulsed illumination, the magnitude of spontaneous polarization varies with time, causing redistribution of the compensation charges through the external circuit. The resulting pyroelectric current can be described by *I_pyro_* = *pA*(*dT/dt*), where *p*, *A*, and *dT/dt* represent the pyroelectric coefficient, active area, and temperature change rate, respectively.

During light ON, rapid light-induced heating decreases the spontaneous polarization and induces redistribution of compensation charges through the external circuit. In the present measurement configuration, this heating-induced pyroelectric response appears as a negative current peak. After the temperature variation becomes stabilized under illumination, the pyroelectric contribution decays, and the current approaches the steady photocurrent level.

When the light is turned OFF, the device returns to the dark state through a cooling process. At this moment, the sign of *dT/dt* is reversed compared with the heating process, and the spontaneous polarization is restored toward its initial state. This temperature-driven polarization recovery induces charge flow in the opposite direction, resulting in a sharp positive current peak. As the cooling process is completed and the transient temperature variation disappears, the pyroelectric current decays, and the device current returns to the dark-current baseline.

**Fig. S2.** Wavelength-dependent effective refractive index of a 10 nm-thick ZnO film deposited on an n-Si substrate under the same deposition conditions used for the top ZnO layer of the device, obtained from spectroscopic ellipsometry using a Cauchy dispersion model. The inset shows the effective refractive index near 550 nm.

**Supplementary Note**

The reduced reflectance under water contact conditions can be explained by the Fresnel reflection at the top interface of the TPV device. **Fig. S2** shows the wavelength-dependent effective refractive index of a 10 nm-thick ZnO film deposited on an n-Si substrate under the same deposition conditions used for the top ZnO layer of the device, obtained from ellipsometric fitting.

For normal incidence, the Fresnel reflectance at an interface can be expressed as:

*R =* $\left| \frac{n_{1}-n_{2}}{n_{1}+n_{2}} \right|^{2}$

where *R* is the reflectance, and *n_1_* and *n_2_* are the refractive indices of the incident medium and the top surface material, respectively. In the air-exposed state, light enters the device through an air/ZnO interface. In the water contact state, this interface is replaced by a water/ZnO interface. Since the refractive index of water is higher than that of air, the refractive-index mismatch between the incident medium and ZnO is reduced under water contact. As a result, the Fresnel reflection at the top interface decreases.

The wavelength-dependent effective refractive index was obtained from ellipsometric fitting of a 10 nm-thick ZnO film deposited on an n-Si substrate. The film was prepared under the same deposition conditions used for the top ZnO layer of the device, and the fitting was performed using a Cauchy dispersion model in the transparent visible region. In this model, the extinction coefficient *k* was assumed to be negligible, and the refractive index *n(λ)* was extracted from the fit. At 550 nm, the fitted effective refractive index was approximately 2. Using *n_air_*=1.00, *n_water_*=1.33, and the experimentally fitted effective *n_ZnO_*≈2 at 550 nm, the calculated reflectance decreases from approximately 11.1% for the air/ZnO interface to approximately 4.0% for the water/ZnO interface.

These values indicate that reducing the refractive-index mismatch between the external medium and the top ZnO layer is an effective strategy for suppressing interfacial reflection and improving optical coupling into the device.

This estimation supports the experimentally observed decrease in reflectance under water contact. Therefore, the enhanced optical coupling under water contact can allow more incident light to enter the TPV heterostructure, contributing to the improved photoresponse of the device.

It should be noted that this Fresnel analysis provides an approximate estimation based on the top-interface reflection. The complete TPV device has a multilayer structure, and the total reflectance can also be affected by interference, absorption, scattering, and the optical constants of each layer. Nevertheless, the comparison between the air/ZnO and water/ZnO interfaces clearly shows that water contact reduces the surface reflection by decreasing the refractive-index mismatch at the device surface.

**Fig. S3.** Comparison of transmittance, reflectance, and absorptance of the TPV device under air and water contact conditions at 365 nm.

**Fig. S4**. Top-view scanning electron microscopy image of the fabricated FTO/ZnO/NiO/AgNW/ZnO device.

**Fig. S5.** Transient current responses of the TPV device measured at optical modulation frequencies from 10 to 100 Hz.

**Supplementary Note**

Well defined transient peaks were reproducibly observed at the light ON and OFF transitions throughout the investigated frequency range. As the modulation frequency increased, the transient peaks appeared more frequently in accordance with the shorter light ON and OFF period, while the characteristic transient response was maintained. This reproducible frequency dependent behavior supports the transient polarization response under repeated optical modulation.

**Fig. S6.** Wavelength dependent transient current responses of the TPV device measured under 365, 410, 490, 530, and 630 nm illumination at 50 Hz.

**Supplementary Note**

The characteristic transient peak was most pronounced under 365 nm excitation and progressively decreased with increasing excitation wavelength. The substantially weakened response at longer wavelengths is consistent with reduced optical absorption and thermal excitation of ZnO, resulting in a smaller temperature dependent polarization change and transient pyroelectric response.

**Fig. S7.** Transient current responses of FTO/NiO/AgNWs and FTO/ZnO/AgNWs control devices measured at an optical modulation frequency of 5 Hz.

**Supplementary Note**

Pronounced transient peaks were consistently observed in the ZnO containing FTO/ZnO/AgNWs device, whereas the ZnO free FTO/NiO/AgNWs control exhibited an almost steady current response without corresponding transient peaks. The selective appearance of the transient response in the ZnO containing device provides material dependent evidence supporting the pyroelectric origin of the dominant transient component.

**Fig. S8.** Transient current responses of the TPV device measured at 0 V and 0.5 Hz under different external conditions: air, 0.1 M NaCl, 1 M NaCl, 2 M NaCl, pH 4, DI water (pH= 7), and pH 11. The NaCl-containing conditions exhibit a progressively enhanced photovoltaic and pyroelectric response with increasing ionic strength, while the pH-controlled measurements exhibit distinct transient responses under acidic and alkaline conditions. These results demonstrate that the transient response is strongly dependent on the ionic and electrostatic environment at the ZnO/liquid interface rather than on physical water contact alone.

 **Fig. S9.** Raw Nyquist plots of the TPV device measured under (a) 0.1 M NaCl, (b) 1 M NaCl, (c) 2 M NaCl, (d) pH 4, and (e) pH 11 conditions at applied biases from −0.2 to 0.1 V. The spectra exhibit distinct bias-dependent impedance responses under each aqueous condition, indicating that both ionic strength and pH modify the overall electrical characteristics of the device. These raw spectra were subsequently analyzed using the equivalent-circuit model to separately evaluate the fitted impedance contributions associated with the ZnO/NiO junction and the ZnO/liquid interface.

**Fig. S10.** Frequency-dependent apparent flat-band potential (*V_FB_*) extracted from Mott–Schottky analysis under pH 4, DI water, and pH 11 conditions. Across the analyzed frequency range, the apparent *V_FB_* consistently followed the order $V_{FB,pH4}<V_{FB,DI}<V_{FB,pH11}$. These relative shifts indicate that the electrostatic state of the ZnO/liquid interface is strongly modified by the pH-controlled aqueous environment. Because the ZnO/NiO junction and ZnO/liquid interface are electrically coupled within the two-terminal device, the extracted values are interpreted as relative apparent *V_FB_* shifts of the coupled device rather than as the intrinsic flat-band potential of isolated ZnO.

**Fig. S11.** Mott–Schottky analysis of the device under air-exposed and water contact conditions. (a–f) Frequency-dependent *1/C^2^–V* plots measured at (a) 250, (b) 100, (c) 50, (d) 25, (e) 20, and (f) 15 kHz. The dashed lines represent the linear fitting regions used to extract the flat-band potential (*V_FB_*). (g) Effective donor density calculated from the Mott–Schottky plots as a function of frequency.

**Supplementary Note :** Frequency-dependent Mott–Schottky analysis

Frequency-dependent Mott–Schottky analysis was performed to further examine the change in the interfacial electrical environment of the FTO/ZnO/NiO/AgNW/ZnO device under air-exposed and water contact conditions. **Fig. S11** (a–f) show the *1/C^2^–V* plots measured at 250, 100, 50, 25, 20, and 15 kHz. Across the measured frequency range, the water contact device exhibited lower *1/C^2^* values than the air-exposed device, indicating an increase in the capacitance response under water contact.

The Mott–Schottky relation can be expressed as

$$\frac{1}{C^{2}}=\frac{2(V-V_{FB}-\frac{kT}{q})}{q\varepsilon_{0}\varepsilon_{r}A^{2}N_{D}}$$

where *C* is the capacitance, *V* is the applied voltage, *V_FB_* is the flat-band potential, *k* is the Boltzmann constant, *T* is the absolute temperature, *q* is the elementary charge, *ε_0_* is the vacuum permittivity, *ε_r_* is the relative dielectric constant, *A* is the active area, and *N_D_* is the effective donor concentration. According to this relation, a lower *1/C^2^* value corresponds to a larger capacitance. Therefore, the reduced *1/C^2^* response under water contact indicates that the space-charge capacitance of the heterojunction is increased.

The effective carrier concentration was estimated from the slope of the linear region of the Mott–Schottky plots using

$$\left| N_{D} \right|=\frac{2}{q\varepsilon_{0}\varepsilon_{r}A^{2}\left| \frac{d\left( \frac{1}{C^{2}} \right)}{dV} \right|}$$

where |*d*(*1/C²*)*/dV*| is the absolute value of the fitted slope. The lower slope magnitude observed under water contact results in a higher effective donor concentration compared with the air-exposed condition. As summarized in **Fig. S11** (g), the calculated |*N_D_*| remained higher under water contact over the measured frequency range, indicating that the water interface enhances the charge response of the device.

In addition, the flat-band potential was obtained from the voltage-axis intercept of the linear fitting line. The water contact device showed a positive shift in *V_FB_* compared with the air-exposed device, suggesting that the electrostatic environment of the junction is modified under water contact. This positive *V_FB_* shift indicates a strengthened built-in electric-field environment, which can assist photogenerated carrier separation under self-powered operation.

Overall, the frequency-dependent Mott–Schottky analysis supports the EIS fitting results. The lower *1/C^2^* response, increased |*N_D_*|, and positive shift in flat-band potential indicate that water contact enhances the capacitance response and modifies the junction electrostatics of the TPV device.

**Fig. S12.** Temperature-dependent photoresponse of the TPV device at *λ*=365nm under water contact at (a) 23 °C, (b) 50 °C, and (c) 70 °C, along with their corresponding thermographic images on the right-hand side. The thermographic images confirm the distinct thermal states under each temperature condition. For the nominal 70 °C condition, 70 °C represents the surrounding water temperature, while 64.8 °C is the local surface temperature measured at the selected position.

**Table S1.** EIS fitting parameters of the TPV device measured at 0 V under air, DI water, and NaCl electrolyte conditions with different ionic strengths. The extracted parameters were obtained using the equivalent-circuit model $R_{s}-(Q_{1}\parallel R_{1})-(C_{\mathrm{EDL}}\parallel R_{2})$. The decrease in *R_s_* and the systematic increase in *C_EDL_* with increasing NaCl concentration indicate reduced series resistance and enhanced interfacial charge-storage behavior at the ZnO/liquid interface.

| Condition | *R_s_* (Ω) | *Q_1_-Y* | *R_1_* (Ω) | *C_EDL_* (µF) | *R_2_* (Ω) | *χ^2^* | *R^2^* |
| --- | --- | --- | --- | --- | --- | --- | --- |
| Air | 37.486 | 0.310 µ | 282.883 | 0.0367 | 8.859 | 1.099007 × 10^-5^ | 0.999972 |
| DI water | 29.254 | 0.623 µ | 396.845 | 0.582 | 142.483 | 8.826478 × 10^-6^ | 0.9999993 |
| 0.1 M NaCl | 27.425 | 2.811 µ | 500.082 | 2.552 | 68.360 | 3.672157 × 10^-5^ | 0.999987 |
| 1 M NaCl | 15.783 | 50.771 µ | 218.272 | 2.871 | 271.591 | 6.587825 × 10^-5^ | 0.999970 |
| 2 M NaCl | 13.770 | 261.647 µ | 349.286 | 3.924 | 238.464 | 2.78 × 10^-4^ | 0.999916 |

**Table S2.** EIS fitting parameters of the TPV device measured at 0 V under pH 4, DI water, and pH 11 conditions. The extracted parameters were obtained using the equivalent-circuit model $R_{s}-(Q_{1}\parallel R_{1})-(C_{\mathrm{EDL}}\parallel R_{2})$. The pH-dependent changes in *R_s_* and *C_EDL_* indicate modification of the ZnO/liquid interfacial electrical environment under acidic and alkaline conditions.

| Condition | *R_s_* (Ω) | *Q_1_-Y* | *R_1_* (Ω) | *C_EDL_* (µF) | *R_2_* (Ω) | *χ^2^* | *R^2^* |
| --- | --- | --- | --- | --- | --- | --- | --- |
| pH 4 | 37.235 | 88.078 µ | 106.230 | 2.269 | 43.609 | 9.627545 × 10^-5^ | 0.999781 |
| DI water | 29.254 | 0.623 µ | 396.845 | 0.582 | 142.483 | 8.826478 × 10^-6^ | 0.9999993 |
| pH 11 | 13.734 | 268.958 µ | 243.767 | 7.675 | 56.198 | 3.451001 × 10^-5^ | 0.999958 |
